# Synthesis of polyvinyl alcohol (PVA) infiltrated MWCNTs buckypaper for strain sensing application

**DOI:** 10.1038/s41598-018-35638-3

**Published:** 2018-11-23

**Authors:** Min Juey Yee, N. M. Mubarak, Mohammad Khalid, E. C. Abdullah, Priyanka Jagadish

**Affiliations:** 1 Department of Chemical Engineering, Faculty of Engineering and Science, Curtin University, 98009 Sarawak, Malaysia; 2 Graphene & Advanced 2D Materials Research Group (GAMRG), School of Science and Technology, Sunway University, No. 5, Jalan Universiti, Bandar Sunway, 47500 Subang Jaya, Selangor Malaysia; 3 Department of Chemical Process Engineering, Malaysia-Japan International Institute of Technology (MJIIT) Universiti Teknologi Malaysia (UTM), Jalan Sultan Yahya Petra, 54100 Kuala Lumpur, Malaysia

## Abstract

Buckypaper (BP)/polymer composites are viewed as a viable option to improve the strain transfer across the buckypaper strain sensor by means of providing better interfacial bonding between the polymer and carbon nanotubes (CNTs). Multiwall carbon nanotubes (MWCNTs) BP/polyvinyl alcohol (PVA) composites were fabricated by a sequence of vacuum filtration and polymer intercalation technique. The optimized conditions for achieving a uniform and stable dispersion of MWCNTs were found to be using ethanol as a dispersion medium, 54 μm ultrasonic amplitude and 40 min sonication time. FTIR analysis and SEM spectra further confirmed the introduction of oxygenated groups (-COOH) on the surface of MWCNTs BP and the complete infiltration of PVA into the porous MWCNTs network. At MWCNTs content of 65 wt. %, the tensile strength, Young’s modulus and elongation-at-break of PVA-infiltrated MWCNTs BP achieved a maximum value of 156.28 MPa, 4.02 GPa and 5.85%, improved by 189%, 443% and 166% respectively, as compared to the MWCNTs BP. Electrical characterization performed using both two-point probe method and Hall effect measurement showed that BP/PVA composites exhibited reduced electrical conductivity. From the electromechanical characterization, the BP/PVA composites showed improved sensitivity with a gauge factor of about 1.89–2.92. The cyclic uniaxial tensile test validated the high reproducibility and hysteresis-free operation of 65-BP/PVA composite under 3 loading-unloading cycles. Characterization results confirmed that the flexible BP/PVA composite (65 wt. %) with improved mechanical and electromechanical properties is suitable for strain sensing applications in structural health monitoring and wearable technology, as an alternative choice to the fragile nature of conventional metallic strain sensors.

## Introduction

Since the discovery by Sumio Iijima in 1991^1^, carbon nanotubes (CNTs) have attracted tremendous attention, owing to their unique structure and remarkable mechanical, electrical and thermal properties. CNTs has a tensile strength of 10–500 GPa, high thermal conductivity of 3000 Wm^−1^K^−1^ ^2^, high surface area of 50 to 1315 m^2^/g ^3^, high electrical conductivity (10^6^–10^7^ S/m)^4^ and high aspect ratio of greater than 1000^5^. These superior properties of CNTs endow themselves with novel technological opportunities as a promising nanofiller material for the reinforcement of composites, in comparison to other carbon nanomaterials. Over the past few decades, significant effort has been focused on the development of high-performance carbon-based polymer composites with improved thermal, mechanical and electrical properties. Reported methods for fabricating carbon-based polymer composite mainly include the incorporation of CNTs in a polymer matrix^6–8^ and infiltrating CNTs buckypaper with polymer solutions^9–12^. Several studies have largely focused on improving CNTs dispersion quality in a polymer matrix using methods such as solution mixing^13–15^, *in-situ* polymerization^16–18^ and melt processing^19–23^. However, the incorporation of CNTs in the polymer matrix typically resulted in poor dispersion due to the low solubility in solvents, strong agglomerating tendency and high viscosity of CNTs/polymer suspension. The high tendency of agglomeration of CNTs in polymer suspension is originated by the inherent van der Waals forces of attraction between CNT nanoparticles and weak interfacial bonding between CNTs and polymer matrices^24,25^. Poor dispersion of CNTs within polymer matrices (e.g. thermosets^26^, thermoplastics^27^ and elastomers^28^) eventually affected their mechanical properties and the corresponding strain sensing performance of CNTs/polymer composites^29^.

Therefore, new technological advancement in polymeric-based buckypaper materials have generated tremendous attention on the vast strain sensing applications. Strain sensors display diverse functionalities in a wide range of applications, ranging from automotive components to medical devices. For instance, human motion detection in wearable technology^7,30^, structural health monitoring for crack detection^31–33^, as well as load and damage sensing in aerospace applications^34,35^. Ideally, strain applied is fully transferred to the CNTs in the strain sensor. However, the buckypaper strain sensor has some interlayer sliding deformation within individual CNTs^36^ and slippage between CNTs in bundles^37^. The slippage among the CNTs in bundles occurs due to weak van der Waals interactions at the junction points of the CNTs, while the interlayer slippage within CNTs occurs due to the sliding of multiple graphene layers of MWCNTs. This may hinder the strain transfer through the strain sensor and affects the performance of strain measurements. Thus, BP/polymer composites are viewed as a viable option to improve the strain transfer across the strain sensor by means of better interfacial bonding. The infiltration of polymer into the porous CNTs network improves interfacial adhesion between the polymer and CNTs, resulting in a stronger bond between the polymer and CNTs with the formation of helical polymer structure^38,39^ In addition, BP/polymer composites have also been considered as a promising option to solve the agglomeration and dispersion issues, producing composites with homogeneous CNTs dispersion, controlled thickness and high CNTs loading of up to 60 wt. %^40^. The polymer intercalation of buckypaper offers an attractive route to promote effective load transfer from the polymer matrix to CNTs reinforcement, leading to improved mechanical properties^41–44^. For instance, Han *et al*.^37^ impregnated both pristine BP (p-BP) and functionalized BP (f-BP) into epoxy (EP) resin solution. Under tensile test, the Young’s modulus, tensile strength and elongation-at-break achieved by p-BP/EP nanocomposite were 11.2 GPa, 125.7 MPa and 1.9%, as compared to that of p-BP with 1.37 GPa, 11.6 MPa and 8.9% respectively. The p-BP/EP nanocomposite showed an improvement of Young’s modulus, tensile strength and elongation-at-break by 718%, 984% and 368% respectively. The Young’s modulus, tensile strength and elongation-at-break achieved by f-BP/EP nanocomposite were 13.8 GPa, 146.1 MPa and 1.3%, as compared to that of f-BP with 3.25 GPa, 27.6 MPa and 3.9% respectively. The f-BP/EP nanocomposite showed an improvement of Young’s modulus, tensile strength and elongation-at-break by 325%, 429% and 200% respectively^41^. Han *et al*.^42^ reported that the Young’s modulus, tensile strength and maximum strain achieved by optimized MWCNTs BP/TPU nanocomposite were 6.02 GPa, 123.2 MPa and 31.3%, as compared to that of pure BP with 1.37 GPa, 11.58 MPa and 8.89% respectively. In a study of BP/polycarbonate composites using infiltration technique, similar results were found by Pham *et al*.^43^, in which the Young modulus and tensile strength increased by about 220 and 300% respectively. Both Young’s modulus and tensile strength increased dramatically by 340% and 960% respectively. Besides, the incorporation of BP into polymer matrices offers an attractive route to minimize the percolation threshold issues. Several studies have reported that the electrical conductivities of BP/polymer nanocomposites were in several orders of magnitude higher (10^1^–10^2^ S/m)^9,10^ than that of conventional CNTs/polymer composites (10^−9^ to 10^−2^ S/m)^14–16,45,46^. This is because more conductive pathways can be formed with a higher CNTs loading of up to 60 wt. %^40^, as compared to CNTs/polymer composites with CNTs loading of up to 5 wt. %^16^.

Based on such background, this research paper emphasizes on the preparation of MWCNTs BP/PVA composite strain sensor by a sequence of vacuum filtration and polymer infiltration technique. The morphologies, mechanical, electrical and electromechanical properties of the composites with different MWCNTs loadings were characterized to validate their feasibility as flexible strain sensors.

## Results and Discussion

Several characterizations were performed to evaluate the suitability and properties of polyvinyl alcohol (PVA) infiltrated MWCNTs buckypaper for strain sensing application. Initally, the covalent functionalization of MWCNTs by liquid phase oxidation was performed to produce oxidized MWCNTs required for the synthesis of MWCNTs buckypaper. Fourier-transform infrared (FTIR) analysis was then performed to validate the attachment of oxygen-containing functional groups on MWCNTs after the chemical functionalization. Zeta potential analysis was carried out to determine the optimized conditions for achieving uniform and stable dispersion of MWCNTs by varying the types of solvents, sonication time and ultrasonic amplitude. Based on the surface modification of MWCNTs buckypaper conducted by infiltrating MWCNTs buckypaper into PVA solution, energy dispersive X-ray (EDX) analysis was performed to identify the elemental composition present on the surface of PVA-infiltrated MWCNTs buckypaper, while field emission scanning electron microscopy (FESEM) was conducted to validate the infiltration of the PVA matrix into MWCNTs buckypaper network. Later, MWCNTs BP/PVA composites with different MWCNTs contents were prepared to investigate the effect of polymer intercalation on the mechanical, electrical and electromechanical properties of PVA-infiltrated MWCNTs buckypaper produced. For the mechanical characterization, tensile tests were performed to evaluate and analyze the mechanical behaviour of the MWCNTs BP and BP/PVA composites based on the stress-strain curves plotted. For the electrical characterization, both two-point probe method using a digital multimeter and four-point probe method using Hall effect measurements were performed to evaluate the electrical performances of the BP/PVA composite. Various electrical properties, such as electrical resistance, resistivity, conductivity, electron density and electron mobility of the MWCNTs BP and BP/PVA composites were determined. For electromechanical characterization, the resistance-strain dependence was examined to determine the gauge factor of the BP/PVA composites, while the cyclic uniaxial tensile test was conducted to investigate the piezoresistive behaviour and reproducibility of the strain sensors during multiple loading-unloading cycles. Finally, all the characterization results were analyzed to determine the most-suitable BP/PVA composite for strain sensing application.

### Characterization of oxidized MWCNTs

#### Fourier Transform Infrared (FTIR) analysis

**Figure 1:**
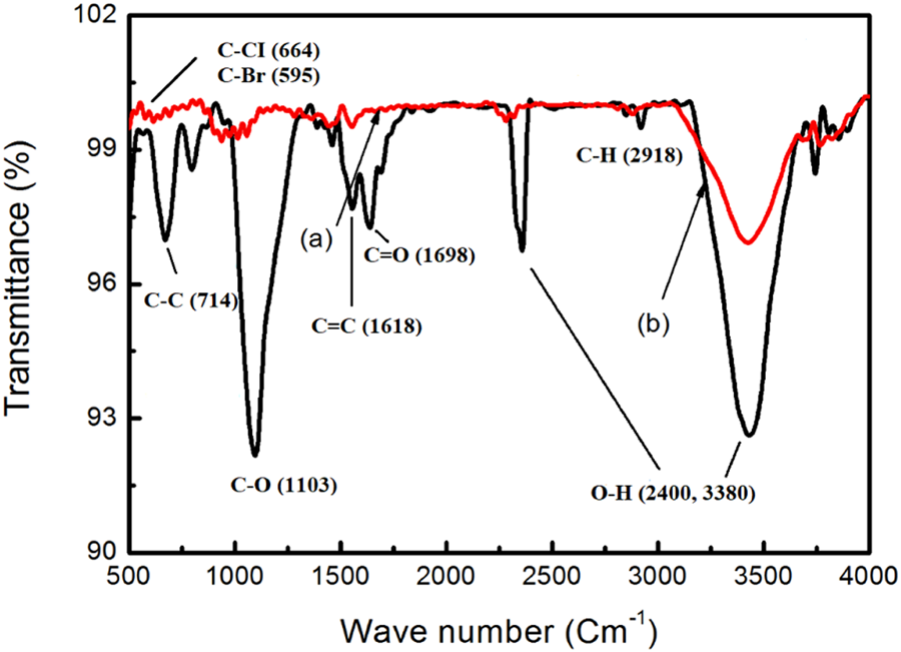
FTIR spectra of (**a**) pristine MWCNTs and (**b**) oxidized MWCNTs.

To provide the evidence of the attachment of oxygen-containing functional groups on MWCNTs after chemical functionalization, a comparison of the FTIR spectra of pristine (p-MWCNTs) and oxidized MWCNTs (o-MWCNTs) were shown in Fig. 1, in the range from 500 to 4000 cm^−1^. FTIR analysis was conducted based on the MWCNTs surface chemistry studies conducted by previous researchers^47–52^. The broad band with a maximum peak located at 3380 cm^−1^ is assigned to the stretching of strongly hydrogen-bonded hydroxyl (O-H) moieties from carboxyl groups. After the surface medication, a higher intensity of the peak at 3380 cm^−1^ indicated the high degree of covalent functionalization of MWCNTs by liquid phase oxidation using H_2_SO_4_/HNO_3_ mixture. The peaks observed at 3176 cm^−1^ and 2918 cm^−1^ were assigned to the asymmetric and symmetric stretching of C-H group. The increased peak intensity at 2400 cm^−1^, after oxidative treatment was also associated with the O-H stretching of the carboxyl groups. Distinct peaks observed at 3380 cm^−1^ and 2400 cm^−1^ have proved that the O-H stretching of carboxyl groups possesses a wide wavelength range, similar to a work reported by Morsy *et al*.^48^. The absorption peak at 1798 cm^−1^ corresponds to the carbonyl (C=O) group, which can be attributed to the stretching vibrations of carboxyl groups (-COOH). The band at 1618 cm^−1^ was related to the backbone of carbon nanotube, alkene (C=C) group, while the region between 1487 and 1333 cm^−1^ was ascribed to the bending of the CH_2_ group. A drastic increase in peak intensity observed at 1103 cm^−1^ was related to the stretching vibrations of more C-O groups, while the band located at 714 cm^−1^ was associated with C-C stretching. The low peaks at 664 cm^−1^ and 595 cm^−1^ corresponded to the negligible existence of C-CI and C-Br halide groups respectively, proving that the oxidation treatment also simultaneously purified MWCNTs. In conclusion, the FTIR measurements have validated the attachments of strongly hydrogen-bonded hydroxyl moieties (O-H) and carbonyl group (C=O) to MWCNTs, which corresponded to the characteristic of carboxyl functional groups (-COOH). A summary of the functional groups assignment of oxidized MWCNTs based on IR spectra was listed in Table 1.

**Table 1:** Assignment of functional groups of oxidized MWCNTs based on IR spectra.

Wavenumber (cm^-1^)	Functional groups
3380	O-H stretching
3176	C-H asymmetric stretching
2918	C-H symmetric stretching
2400	O-H stretching
1698	C=O
1618	C=C
1487-1333	CH_2_ bending
1103	C-O stretching
714	C-C stretching
664	C-CI
595	C-Br

#### Zeta potential

**Figure 2:**
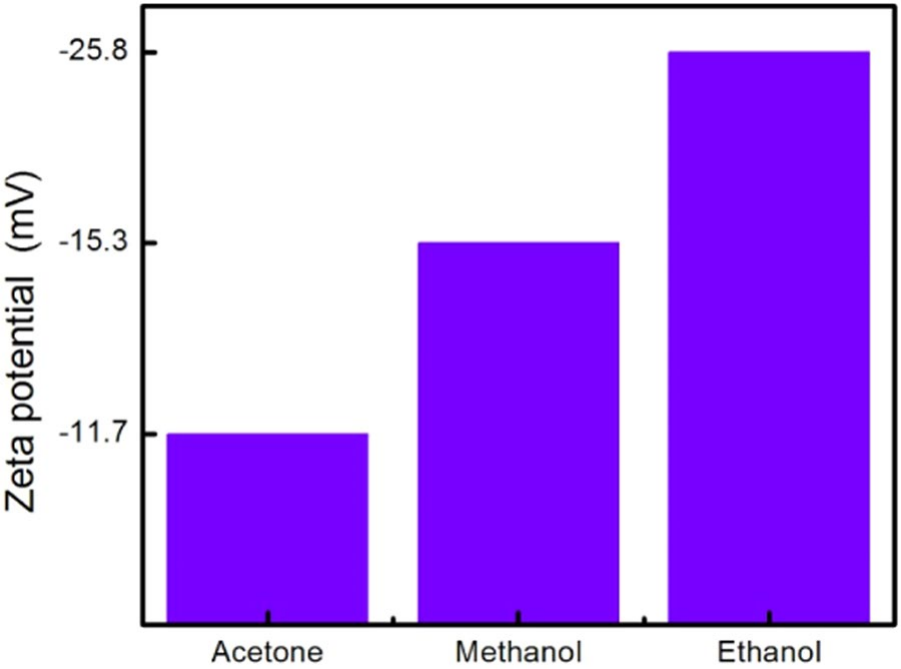
Zeta potential of o-MWCNTs dispersed in various solvents under same sonication conditions (40 min sonication at 60% ultrasonic amplitude).

A well-dispersed MWCNTs aqueous suspension is crucial for obtaining extraordinary MWCNTs film with superior electrical properties^53^ and mechanical properties^29^. In this research, the optimized conditions of excellent oxidized MWCNTs (o-MWCNTs) dispersion was found to be using ethanol as a dispersion medium, at an ultrasonic amplitude of 54 μm and a sonication time of 40 min. During the sonication process, MWCNTs are gradually exfoliated and disentangled from MWCNTs bundles and aggregates. The dispersibility of surface-modified MWCNTs with carboxylic groups in polar ethanol was significantly enhanced, owing to the combination of polar-polar affinity and electrostatic repulsion^54^. Electrostatic repulsion is originated from the zeta potential analysis of MWCNTs with carboxylic anion groups. Zeta potential analysis was performed using dynamic light scattering (DLS) technique in combination with the influence of an electric field (electrophoresis) to further validate the dispersion stability and surface charge. As shown in Fig. 2, the zeta potential of o-MWCNTs in ethanol, methanol and acetone are −25.8, −15.3 and −11.7 mV respectively. A negative sign of zeta potential indicates the negative surface charge of MWCNTs nanoparticles due to the presence of -COOH carboxyl groups. The high magnitude of zeta potential resembles the high degree of electrostatic repulsions between adjacent o-MWCNTs nanoparticles in the dispersion medium. The high zeta potential of −25.8 mV indicates that o-MWCNTs were dispersed individually in ethanol without any visible aggregates, resembling a higher dispersion stability. This is because the electrical double layer, consisting of rigid layer attached the particles (Stern layer) and diffuse layer produces the electrostatic repulsion to overcome the van der Waals intra-particle attraction, allowing the o-MWCNTs to be homogeneously dispersed. The zeta potential results obtained are within an acceptable range as the suspensions with a zeta potential value of less than −15 mV or more than 15 mV are considered to be stable due to electrostatic repulsion mechanism^55^, whereas a high absolute value of 40 mV is regarded as an indication of high-quality MWCNTs dispersion stability in solvents^56^. In conclusion, the electrostatic repulsion between the relatively charged MWCNTs surfaces is crucial for the stabilization of the MWCNTs clusters in aqueous solution.

#### Morphological examination

**Figure 3:**
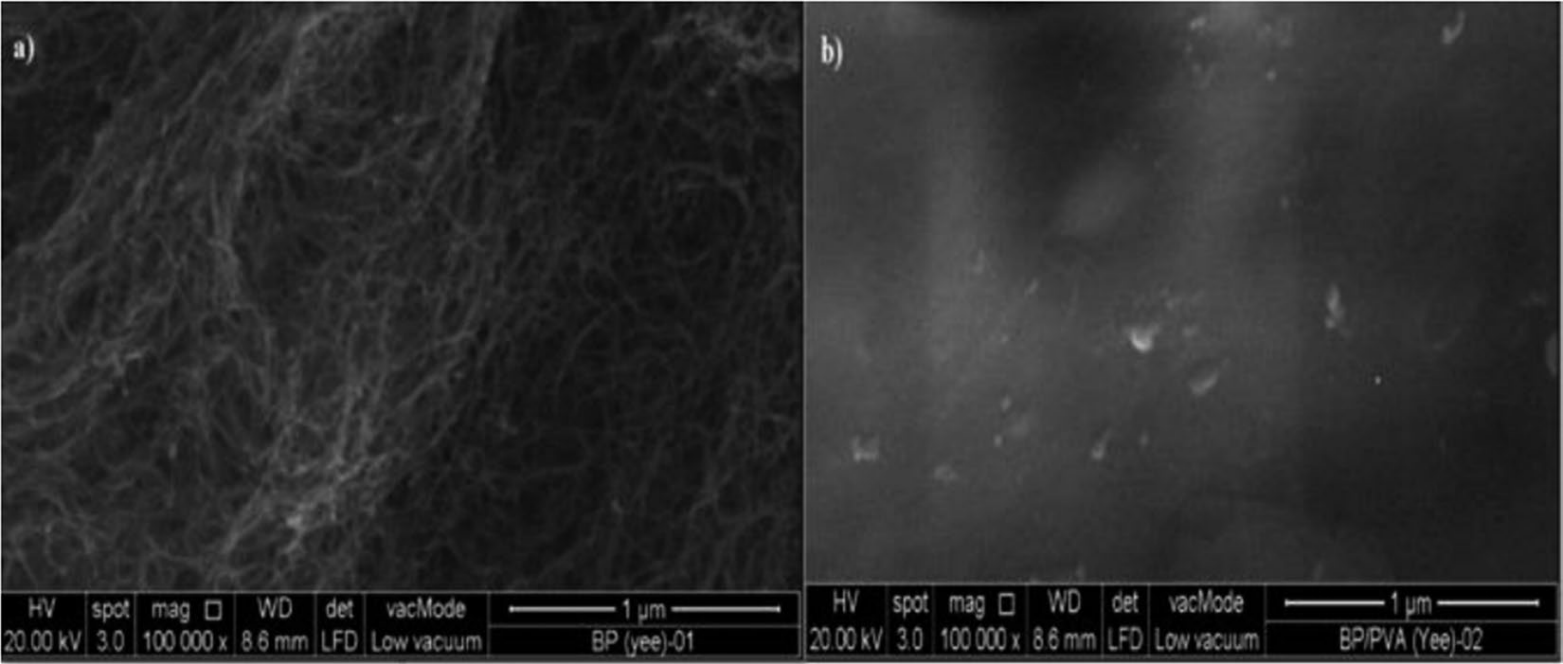
SEM surface images under a magnification of 100,000 × (1 μm): (**a**) MWCNTs buckypaper and (**b**) 65-BP/PVA composite.

SEM surface images of buckypaper sample are presented quantitatively in Fig. 3 to show the morphology and uniformity of buckypaper samples. In Fig. 3(a), surface morphology of MWCNTs buckypaper exhibited dense morphologies with minimal agglomerates. Very few visible pores were observed, even at a high magnification of 100,000×. This indicated that the randomly-oriented MWCNTs bundles were spread homogeneously throughout the surface of buckypaper films, after the surface modification of MWCNTs. The uniform surface dispersion of buckypaper fabricated allows the effective transfer of electrons in the buckypaper, which gives rise to the strain sensing capability of buckypaper.

Based on the surface morphology of the 65-BP/PVA composite shown in Fig. 3(b), no visible pores were observed throughout the surface of 65-BP/PVA composite, verifying excellent infiltration of PVA solution into MWCNTs buckypaper network, similar to a previous work^57^. Uniformly distributed MWCNTs networks were formed in the composites with high MWCNTs loading, indicating the homogeneous distribution of MWCNTs in the PVA matrix. The homogeneity of 65-BP/PVA film was successfully maintained by a sequence of vacuum filtration process and PVA infiltration. Also, no obvious interfacial defects or delaminations, which may affect the stress transfer from the PVA polymer to the BP film were observed. In addition, the good wettability of PVA on MWCNTs facilitated the effective infiltration of PVA into the gaps of the porous network of MWCNTs, forming the relatively defect-free composite film. In conclusion, the coupled effect of the strong PVA-MWCNTs interfacial interaction, good wettability, and long infiltration period (24 hr) gives rise to the low porosity of PVA-infiltrated MWCNTs buckypaper.

#### Elemental composition

Energy dispersive X-ray (EDX) analysis of the surface of MWCNTs buckypaper and 65-BP/PVA buckypaper was performed to identify their elemental compositions. As illustrated in Table 2, the high intensity of C-peak at ~0.25 keV confirmed that MWCNTs buckypaper contains a high mass fraction of carbon atoms (89.80 wt. %), while the intensity of O-peak at ~0.5 keV validated the presence of oxygen elements (8.72 wt. %) in MWCNTs buckypaper, owing to the oxidative treatment of MWCNTs using H_2_SO_4_/HNO_3_. The low intensities of Al-peak, Si-peak and Ca-peak signified that only a small amount of metal catalyst residues entrapped within the carbon layers of MWCNTs buckypaper, after simultaneously performing liquid phase oxidation. The presence of sulphur and the non-metallic element might be due to the addition of sulphuric acid, H_2_SO_4_ during oxidation and its function as MWCNTs growth promoter during the synthesis of MWCNTs by chemical vapour deposition^58^. 65-BP/PVA composite exhibited a higher mass fraction of oxygen element (25.95 wt. %) than that of MWCNTs buckypaper (8.72 wt. %), due to the infiltration of PVA, containing hydroxyl groups (-OH) and the existence of carboxyl groups (-COOH) on the surface of PVA-infiltrated MWCNTs buckypaper after the oxidative treatment.

**Table 2:** EDX analysis of MWCNTs buckypaper and 65-BP/PVA composite.

Elements	Elemental composition (%)	
	MWCNTs BP	65-BP/PVA composite
C	89.80	73.17
O	8.72	25.95
Al	0.42	0
Na	0	0.87
Si	0.12	0
S	0.33	0
CI	0.48	0
Ca	0.14	0

#### Mechanical properties

**Figure 4:**
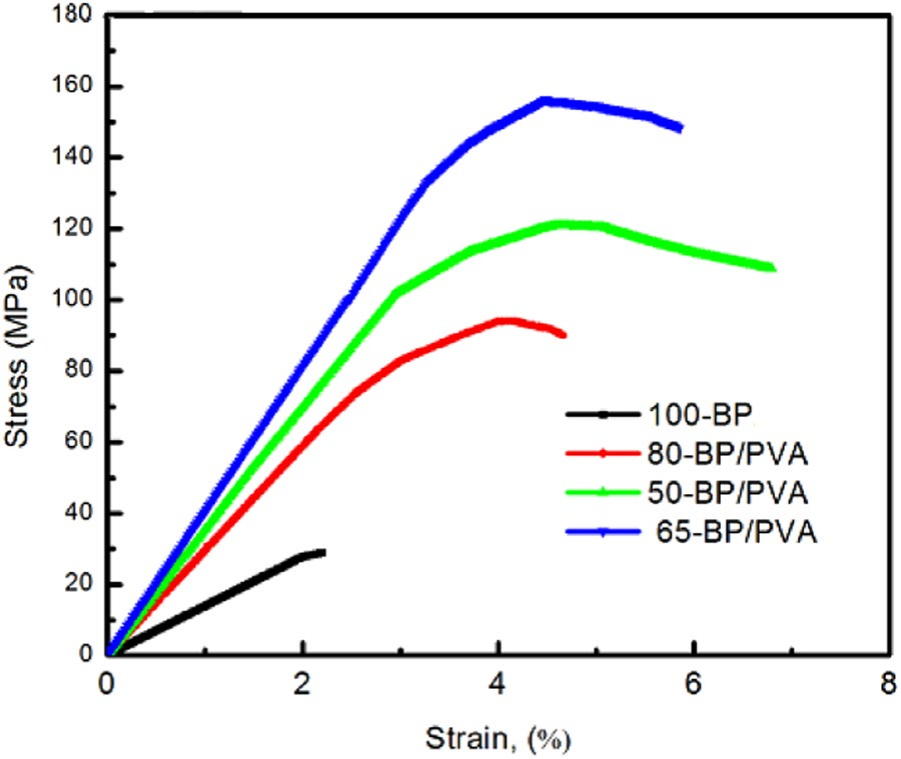
Typical uniaxial tensile stress–strain curves of the MWCNTs buckypaper and BP/PVA composites.

Tensile tests were performed to evaluate the mechanical behaviour of the MWCNTs BP (100 wt.%) and MWCNTs BP/PVA composites with 50 wt. %, 65 wt. % and 80 wt. % MWCNTs content respectively. The typical stress-strain curves for MWCNTs buckypaper and BP/PVA composites are depicted in Fig. 4, whereby the slope in the elastic deformation region represents Young’s modulus. In this case, the tensile strength represents the maximum stress that BP samples can withstand under stretching before failure, while Young’s modulus (modulus of elasticity) represents the stiffness and ability of BP samples to withstand deformation under tension. The strain-to-failure (or elongation-at-break) denotes the ratio of changed length and initial length after the breakage of BP samples.

As shown in Fig. 4, the stress-strain curves of BP samples are divided into two characteristic regions, which are elastic region and inelastic region. The stress-strain curve for 100-BP (black line) showed the flattest slope in the elastic region (ε ~ 2%), implying the low tensile strength (28.77 MPa) and Young’s modulus (1.39 GPa) achieved. 100-BP sample (black line) showed a limited linear range, due to the contact separation and slippage between MWCNT bundles, which are entangled together by weak van der Waals interactions. Above the elastic limit at a stress of 27.8 MPa, MWCNTs buckypaper (or 100-BP) experienced inelastic deformation, resulting in permanent irreversible changes in the conductive MWCNTs network, even when the tensile load is removed. MWCNTs buckypaper was unable to recover to its original shape, due to the breakage of bonds between carbon atoms. The further increase in tensile load eventually led to the fracture of MWCNTs buckypaper at the narrow portion. Besides, a relatively narrow range of the stress-strain curve of 100-BP implied that low strain-to-failure (2.2%) was obtained, while an absence of the necking region in the stress-strain curve indicated that the MWCNTs buckypaper produced was brittle and less flexible.

Moreover, it was observed that the BP/PVA composites exhibited linear elastic deformation, followed by non-linear inelastic (or plastic) deformation and necking until the fracture of buckypaper samples occurred. During necking, strain localizes disproportionately in the small necking region of BP/PVA composites and a relatively lower tensile stress is required for the further elongation of the composite. The cross-section in the necking region of BP/PVA composites gradually decreases until the composites break abruptly. All of the stress-strain curves for MWCNTs BP/PVA composites generally showed steeper linear slopes in their elastic deformation regions (ε ~ 2.5–3%) respectively, as compared to that of MWCNTs BP (ε ~ 2%). Particularly, the stress-strain curve of 65-BP/PVA composite (blue line) displayed the steepest slope throughout the elastic region, evidencing the highest Young’s modulus (4.02 GPa) and tensile strength (156.28 MPa) obtained. Both of the 80-BP/PVA composite (red line) and 50-BP/PVA composite (green line) exhibited slightly flatter slopes of the stress-strain curve in the elastic deformation range. The broad range of the stress-strain curve of 50-BP/PVA composite indicated that high strain-to-failure (6.8%) was obtained. This simply means that the 50-BP/PVA composite can be elongated up to 106.8% of its original length before the fracture of the composite occurs. A summary of the mechanical properties of the MWCNTs buckypaper and BP/PVA composites is presented in Table 3.

**Table 3:** Summary of mechanical properties of MWCNTs buckypaper and BP/PVA composites with different MWCNTs loading.

Sample name	Tensile strength (MPa)	Young’s modulus (GPa)	Strain-to-failure (%)
100-BP	28.77	1.39	2.20
80-BP/PVA	93.98	2.91	4.66
65-BP/PVA	156.28	4.02	5.85
50-BP/PVA	120.93	3.43	6.80

The tensile strength, Young’s modulus and strain-to-failure of MWCNTs BP (or 100-BP) are 28.77 MPa, 1.39 GPa and 2.2% respectively. Both of the tensile strength and Young’s modulus of MWCNTs BP were noticeably higher than the reported values of 0.68–7.5 MPa and 426–785 MPa in the literature respectively^57,59,60^, a likely result of the comparatively higher MWCNTs content used. Nonetheless, the tensile strength and Young’s modulus of 100-BP were significantly lower than that of the BP/PVA composites, possibly due to the interlayer slippage within MWCNTs and slippage between MWCNTs bundles^36^. Interlayer slippage is inevitable as it is an inherent structural property of MWCNTs. Interlayer slippage within MWCNTs occurs due to the sliding of graphene layers of MWCNTs, while the slippage between MWCNTs bundles occurs due to the weak van Der Waals attraction between MWCNTs^37^. In addition, the poor mechanical properties of the 100-BP sample can be attributed to the aggregation of MWCNTs at high loading (100 wt.%) and the lower energy required to de-bridge the MWCNTs bundles during crack formation and crack propagation^61^.

The tensile strength of PVA-infiltrated MWCNTs buckypaper samples (or BP/PVA composites) produced ranged from 93.98–156.28 MPa, while the Young’s modulus ranged from 2.1–4.02 GPa and the strain-to-failure ranged from 4.66–6.80%, varied according to the MWCNTs content. The overall results revealed that PVA-infiltrated MWCNTs buckypapers have higher tensile strength, Young’s modulus and strain-to-failure, as compared to MWCNTs BP. This enhancement of mechanical properties was probably due to the improved interfacial interactions between the PVA matrix and MWCNTs. During the soaking process, the PVA solution diffused into the buckypaper and easily interacted with the oxidized MWCNTs. In addition, hydrogen bonding between the hydrophilic oxygen-containing functional groups (e.g. -COOH, -OH, C=O) may also contribute to the improved interactions between PVA hydroxyl groups and MWCNT carboxyl groups. When MWCNTs buckypaper was impregnated with PVA solution, the hydroxyl groups (O-H) of PVA can interact with hydroxyl (O-H), carbonyl (C=O) and carboxyl (-COOH) groups on the MWCNTs surface. Thus, the strong interfacial adhesion between functionalized MWCNTs and PVA matrix was achieved, greatly enhancing the dispersion and the corresponding mechanical properties of the composites^62^.

There was a huge variation in the mechanical properties among the BP/PVA composites. Table 3 showed that the strain-to-failure of the composites decreases, when higher MWCNTs content is present in the composites. This is due to an increased restriction of PVA chain mobility under the presence of MWCNTs. The tensile strength and modulus do not show monotonic trend with the MWCNTs content. Among the BP/PVA composites, 65-BP/PVA composite achieved the highest tensile strength (156.28 MPa) and Young’s modulus (4.02 GPa), due to the possible complete infiltration of PVA into the MWCNTs network. The porous MWCNTs network was completely filled with soluble PVA solution, which resulted in an improved interfacial bonding between MWCNTs and PVA matrix and the corresponding better mechanical properties. The tensile strength of 65-BP/PVA composite produced by the soaking method (156.28 MPa) was higher than the reported tensile strength of MWCNTs/PVA composites (27.9–51.1 MPa) produced from solution mixing method^63,64^. Besides, Young’s modulus of 65-BP/PVA composite (4.02 GPa) was also remarkably higher than the reported values of MWCNTs BP/PVA ranged from 0.25 to 1.37 GPa, produced from similar soaking method^57,65^. However, the further addition of PVA polymer onto the MWCNTs buckypaper caused Young’s modulus to decrease from 4.02 to 3.43 GPa respectively. This effect may be attributed to the significant decrease in the tensile strength from 156.28 to 120.93 MPa, as a result of higher PVA content and lower MWCNTs content (50 wt. %) in the composite. When a higher amount of PVA solution was added, PVA grafted to the surface of MWCNTs BP quickly, causing the pores at the surface to block by part of the insoluble PVA hydrogel. The presence of non-uniform localized PVA deposit on the surface of BP/PVA composite restricted the diffusion of soluble PVA solution into the MWCNTs BP. These phenomena might lead to an incomplete infiltration of PVA into the inner core of the MWCNTs BP and also resulted in a limited improvement in mechanical properties for 50-BP/PVA composite.

In conclusion, the incorporation of PVA polymer into MWCNTs BP can dramatically enhance the mechanical properties of the composites. Based on the mechanical characterization, the MWCNTs content of 65 wt. % was found to be the optimized MWCNTs loading for the synthesis of flexible PVA-infiltrated MWNCTs buckypaper for strain sensing application. The Young’s modulus, tensile strength and strain-to-failure of 65-BP/PVA composite improved by 443%, 189% and 166% respectively, as compared to MWCNTs BP. The improvement of mechanical properties of 65-BP/PVA composite was evidenced by the intensive intermolecular-level interactions between MWCNTs and PVA chain molecules, as well as the high degree of PVA infiltration into the porous MWCNTs network. The improved interfacial adhesion between PVA and MWCNTs allows the effective load transfer from PVA matrix to MWCNTs, further verifying the feasibility of 65-BP/PVA composite produced for strain sensing application.

### Electrical properties

#### Two-point probe method

**Figure 5:**
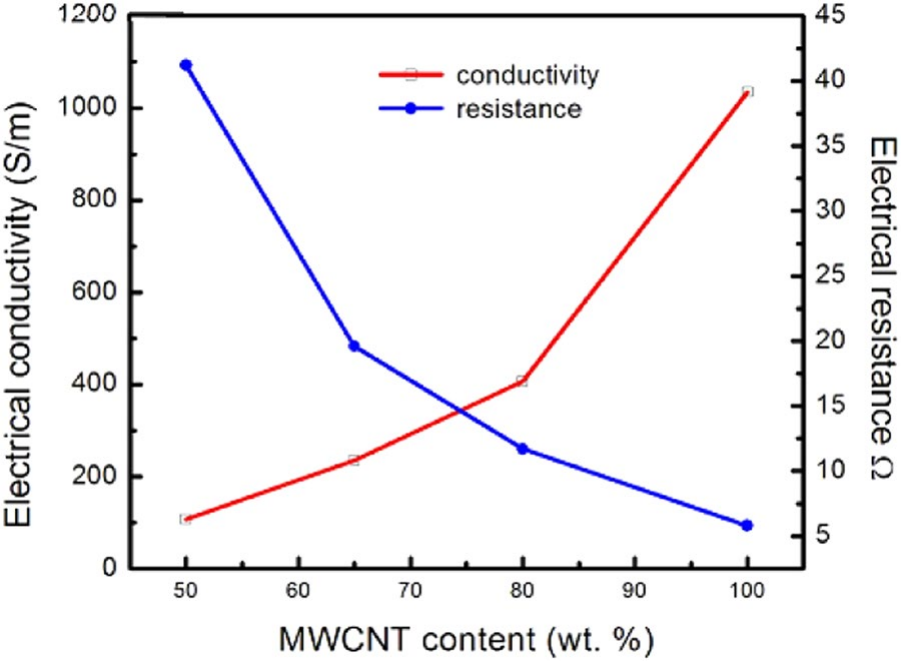
Electrical resistance and conductivity of buckypaper samples as a function of MWCNTs content in the composites using two-point probe method.

Buckypaper has an electrical resistance that encompasses three major components: the contact resistance between the MWCNTs, the tunnelling resistance between the neighbouring MWCNTs and intrinsic resistance of the individual MWCNTs^66,67^. A dense network of conductive MWCNTs in buckypaper promotes a seamless flow of current through the buckypaper^68^. To evaluate the electrical performance of buckypaper strain sensor, DC electric measurement of BP samples was performed using digital multimeter based on the concept of two-point probe method, in accordance to ASTM D4496-13 standard. The trend of electrical resistance and conductivity of the samples with respect to MWCNTs content in the composites using two-point probe method was presented in Fig. 5 below.

It can be noticed that a non-linear trend of the conductivity curve was obtained, whereby the electrical resistance of samples declines and the conductivity increases exponentially, as the MWCNTs content in the composites becomes higher. The electrical conductivity of oxidized MWCNTs BP (100 wt. %) was found to be 1.03 × 10^3^ S/m, lied within the range of BP conductivity (6.6 × 10^2^ to 2 × 10^4^ S/m) reported in the literature ^41,57,69,70^. For example, Han *et al*.^41^ impregnated both pristine BP (p-BP) and functionalized BP (f-BP) nanocomposites with epoxy (EP) resin. Comparing the BP/EP and neat BP, the electrical conductivity of p-BP/EP obtained decreased by 200% from 5.7 × 10^3^ S/m to 1.9 × 10^3^ S/m, while the electrical conductivity of f-BP/EP obtained decreased by 218% from 2.1 × 10^3^ S/m to 6.6 × 10^2^ S/m^41^. DeGraff *et al*.^70^ fabricated printable low-cost and flexible MWCNTs-BPs strain sensor with an electrical conductivity of 2 × 10^4^ S/m.

The high conductivity of MWCNTs BP (100 wt. %) was attributed to the uniform dispersion and the dense packing of MWCNTs buckypaper, leading to better contacts among the MWCNTs. However, the electrical conductivity of oxidized MWCNTs BP obtained may be slightly affected by the surface modification of MWCNTs. Marcelino *et al*.^71^ reported that the electrical conductivity of the surface-modified MWCNT composite was lower than that of the unmodified MWCNTs composites with 510 S/m. This effect was ascribed to increased defects in the lattice structure of C-C bonds on the MWCNTs surface as a result of the acid treatment. Besides, Thakur and his coworker reported that chemical functionalization disrupted the extended π-conjugation of covalently-functionalized MWCNTs composite and hence reduced their electrical conductivity^72^. Nonetheless, the electrical conductivity of MWCNTs buckypaper was still higher than that of PVA-infiltrated MWCNTs buckypapers fabricated using both methods, as shown in Table 4 and Table 5 respectively.

**Table 4:** Summary of electrical properties of PVA-infiltrated MWCNTs buckypaper samples measured using two-point probe method.

Sample name	Film thickness (mm)	Average electrical resistance, R (Ω)	Bulk resistivity, ρ_b_ (Ωm)	Electrical conductivity, σ (S/m)
100-BP	0.50	5.81	9.67 × 10^−4^	1.03 × 10^3^
80-BP/PVA	0.63	11.72	2.46 × 10^−3^	4.07 × 10^2^
65-BP/PVA	0.65	19.60	4.68 × 10^−3^	2.35 × 10^2^
50-BP/PVA	0.68	41.21	9.34 × 10^−3^	1.07 × 10^2^

**Table 5:** Summary of electrical properties of buckypaper samples measured using the four-point probe method.

Sample name	Electron density, N_e_ (cm^−3^)	Electron mobility, μ_e_ (cm^2^/Vs)	Electrical resistance, R (Ω)	Bulk resistivity, ρ_b_ (Ω m)	Electrical conductivity, σ (S/m)
100-BP	1.23 × 10^21^	1.53 × 10^−1^	6.63	3.30 × 10^−4^	3.03 × 10^3^
80-BP/PVA	9.72 × 10^20^	5.25 × 10^−2^	12.32	1.22 × 10^−3^	8.17 × 10^2^
65-BP/PVA	4.79 × 10^20^	9.40 × 10^−2^	15.04	1.39 × 10^−3^	7.21 × 10^2^
50-BP/PVA	5.44 × 10^20^	7.87 × 10^−2^	25.91	1.46 × 10^−3^	6.86 × 10^2^

From Table 4, it can be observed that the polymer intercalation of MWCNTs buckypaper has increased the thickness, electrical resistance and resistivity of the composites. As compared to MWCNTs buckypaper, BP/PVA composites exhibited reduced electrical conductivity (up to 90%), due to the presence of a transparent PVA layer on the surface of the composite during the infiltration process. During the infiltration process, PVA grafted to the surface of buckypaper quickly and filled up the pores of buckypaper. As the amount of PVA solution increases, a significantly thicker layer of transparent PVA surface deposition was formed, resulting in a higher surface resistance measurement. This led to a higher value of bulk resistivity and a corresponding lower conductivity. In other words, the highest bulk resistivity and the corresponding lowest electrical conductivity observed for 50-BP/PVA composite is an indication of a thicker layer of PVA surface deposit, as compared to other BP/PVA composites.

This phenomenon can be further proved by the slight increase in the thickness of BP/PVA composites from 0.63 to 0.68 mm, as a result of more PVA clinging to the surface of MWCNTs buckypaper. In addition, the decline in electrical conductivity observed among the MWCNTs BP/PVA composites, may be attributed to the high electrical insulating behaviour of PVA polymer. The attachment of electrically insulating PVA at the interface of the composite increases the surface and bulk resistivity, which results in a lower conductivity. The penetration of electrically insulating PVA into the porous MWCNTs network fills the gaps between adjacent MWCNTs, which may cause the reduced availability of electrically conductive pathways and the increase in contact and tunnelling resistances between adjacent MWCNTs.

#### Four-point probe method (Hall effect measurement)

**Figure 6:**
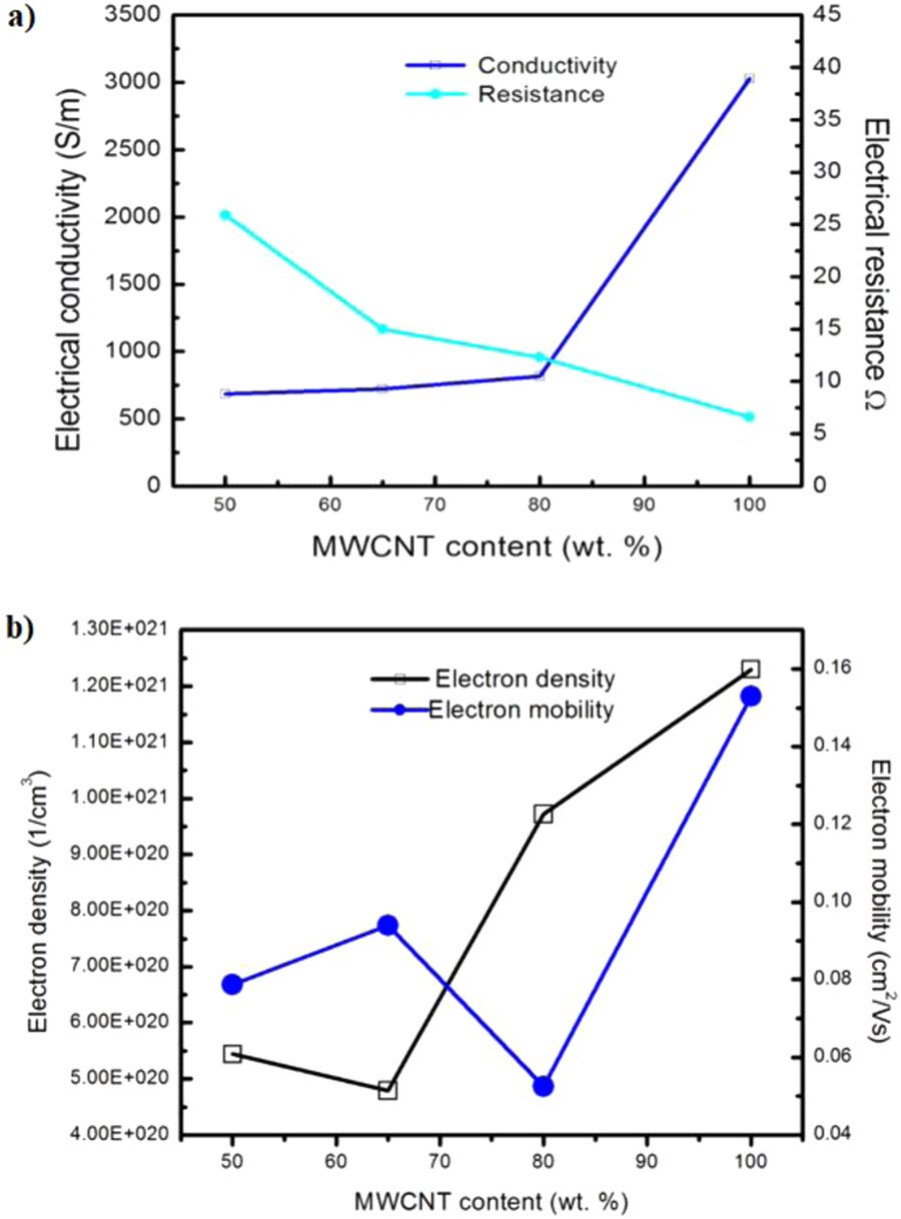
Electrical properties of BP samples as a function of MWCNTs content using four-point probe method: (**a**) Electrical resistance and conductivity (**b**) Electron mobility and electron density.

The electrical properties (e.g. charge carrier (e.g. ions, electron or holes) density, electron mobility, resistivity and conductivity) of the MWCNTs BP samples were further investigated by a four-point probe technique using a van der Pauw Hall effect measurement system (Ecopia HMS 3000, Korea). The results of electrical characterization using Hall effect measurement are depicted in Fig. 6 respectively.

As shown in Fig. 6(a), the electrical conductivity of buckypaper samples exhibited a positive trend, while the electrical resistance of buckypaper samples exhibited a negative trend when subjected to the increment of MWCNTs content in the composite. High MWCNTs content in the composite implies that a correspondingly lower amount of PVA solution is present in the composite. Overall, the trends obtained were similar to that of using a two-point probe method. A summary of the electrical properties of buckypaper samples measured using the four-point probe method was also tabulated in Table 5. As shown in Fig. 6(b) and Table 5, the highest electrical conductivity obtained in MWCNTs buckypaper (3.03 × 10^3^ S/m) was ascribed to the higher electron density (1.23 × 10^21^ cm^−3^) and electron mobility (1.53 × 10^−1^ cm^2^/Vs), as well as a higher content of MWCNTs (100 wt. %). Higher content of MWCNTs creates a denser network of electrical conducting pathways, which result in a lower bulk electrical resistivity and higher electrical conductivity. The high conductivity of 100-BP obtained proved that the electronic structure of MWCNTs buckypaper (or 100-BP) produced was well-preserved. On the other hand, the infiltration of PVA has caused the electronic conductivity to be generally lower than the 100-BP without infiltration, as shown in Fig. 6(a). The electrical conductivities of PVA-infiltrated BPs are within an order of 10^2^ S/m, which is possibly acceptable for the strain sensing application.

Besides, the carrier densities of all samples lied within the range of 10^20^–1021 cm^−3^, which were found to be higher than the carrier density reported in earlier studies (10^18^–10^19^ cm^−3^)^73^. Among the BP/PVA composites, 65-BP/PVA composite attained the highest electron mobility (9.40 × 10^−2^ cm^2^/Vs), but with the lowest carrier (electron) density (4.79 × 10^20^ cm^−3^). This indicated that the low-density electrons can move freely through the composite at a faster rate, leading to a better strain sensing performance. Thus, the electrical conductivity of buckypaper samples was found to be proportional to the product of electron density, N_e_ and electron mobility, μ_e_, in which q is the electric charge (1.6 × 10^−19^ C), as shown in the Eq. 1.

1 $$\sigma =Ne\times \mu e\times q$$

In conclusion, both methods have proven that the electrical conductivity of MWCNTs buckypaper decreased when subjected to the polymer infiltration of polyvinyl alcohol (PVA) solution. The electrical conductivity of PVA-infiltrated MWCNTs buckypaper was dominated by the thickness of the electrically insulating PVA layer. The thicker the PVA layer, the lower the conductivity. Thus, the control of the PVA layer entails the optimization of the PVA infiltration process, including the amount and concentration of the PVA solution, as well as the soaking time. The high degree of polymer intercalation has led to a decrease in electrical conductivity of PVA-infiltrated MWCNTs buckypaper from 4.07 × 10^2^ to 1.07 × 10^2^ S/m (2-point probe method) and from 8.17 × 10^2^ to 6.86 × 10^2^ S/m (4-point probe method) at room temperature, up to 50 wt. % MWCNTs. Noticed that in general, a higher electrical conductivity of BP samples was obtained using a 4-point probe method, which may be attributed to the elimination of contact resistances from the measurement. Nonetheless, MWCNTs BP/PVA composite fabricated are still considered as highly conductive, outperforming the conventional MWCNTs/polymer composites with low electrical conductivities ranged from 10^−9^ to 10^−2^ S/m^14–16,45,46^. Particularly, both 80-BP/PVA (4.07 × 10^2^ S/m) and 65-BP/PVA composites (2.35 × 10^2^ S/m) achieved higher electrical conductivity than the reported MWCNTs BP/TPU composite (1.1 × 10^2^ S/m), with similar soaking method^10^. Considering the differences in the materials and preparation methods, the conductivity values obtained are within the experimental accuracy.

#### Electromechanical response

**Figure 7:**
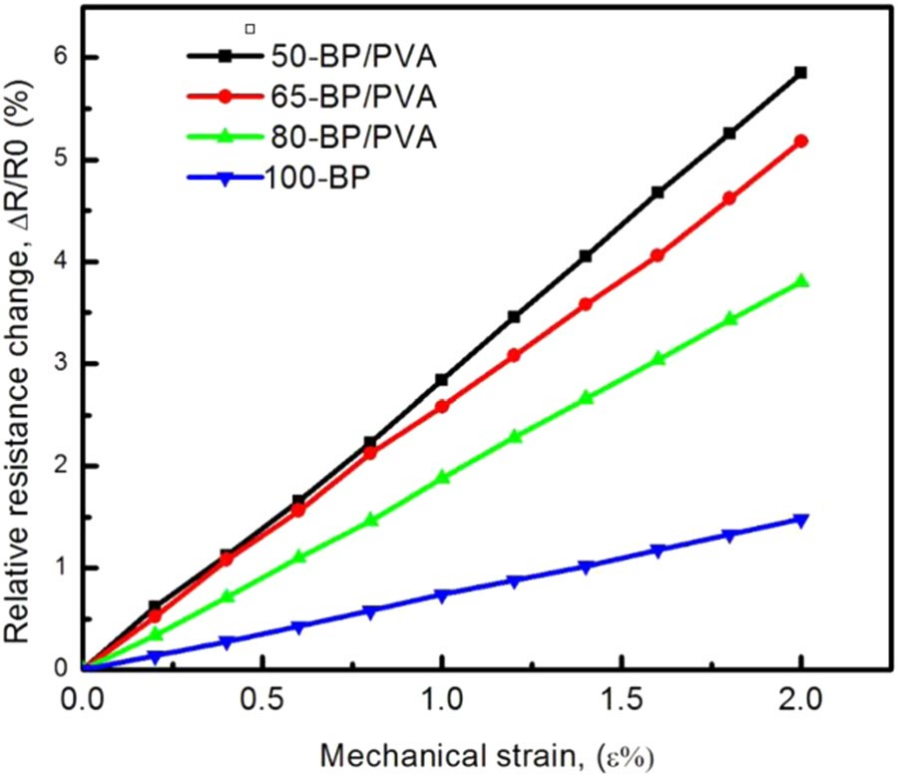
Relative resistance change as a function of mechanical strain of BP/PVA composites in the elastic zone.

The resistance–strain dependence of samples was examined to evaluate the potential of the BP/PVA composite as a strain sensor. For the study of piezoresistivity effect, several BP samples infiltrated with different amount of PVA solution were fabricated. The electromechanical properties of the composite were evaluated in the elastic range (ε ~ 2.0%). Figure 7 presents the relative resistance change (ΔR/R_0_, R_0_ is the original resistance in the unloaded condition and ΔR is the instantaneous change in resistance) as a function of mechanical strain of BP/PVA composites. MWCNTs buckypaper prepared (denoted as 100-BP) was included for the comparison purpose.

As shown in Fig. 7, the relative resistance changes of all samples were found to increase linearly with the strain applied. The increment of resistance change with strain can be attributed to the reduction of the conductive MWCNTs network density and the increase of inter-tube distances induced by tensile strains. Upon tension, the cross-sectional area of the samples decreases and the length of the samples increases. The continual stretching of the BP samples increase in the gap between MWCNTs bundles and the decrease in a number of conductive paths in the BP network, resulting in an increase in electrical resistance. In fact, early studies have suggested that under structural deformation of MWCNTs network, the transformation of local bonding configuration has changed from sp^2^ to nearly sp^3^ configurations^22,74^. In the elastic deformation zone for strain up to 2.0%, the relation between ΔR/R_0_ and ε is fairly linear, thus the gauge factor can be calculated as the slope of the curve. When the mechanical deformation exceeds 2.0%, a non-linear trend is expected, in which the slope increases gradually until film fracture. At the film fracture, the discontinuity in ΔR/R_0_ occurs, as the MWCNTs network in the composite is disrupted and the samples fail to recover due to the structural degradation caused by large strain.

By fitting straight lines to the resistance–strain curves, the gauge factors (a measure of sensor’s sensitivity) of MWCNTs buckypaper and their composites were determined from the linear slopes of the curves. As illustrated in Fig. 7, the 50-BP/PVA (black line) composite demonstrated the steepest line, with the highest ΔR/R_0_ obtained at the same strain level, indicating that the highest sensitivity of composite was achieved, as compared to the other samples. 100-BP (blue line), 65-BP/PVA (red line) and 80-BP/PVA (green line) composites exhibited similar linear piezoresistive behaviour, but with flatter slopes respectively. In contrast, the non-linear trend of the piezeresistive response of MWCNTs/polymer composite under high deformation has also been reported in the literature. Tadajaluru and his coworkers observed the nonlinearity of the piezeresistive response of MWCNTs/natural rubber composite film, due to non-uniform deformation of the conducting layers on the natural rubber^75^. Hu *et al*.[ref] reported that the resistance increased non-linearly under large mechanical deformation of 6000 με, due to the increased tunnelling resistance between neighbouring MWCNTs. The non-linear behaviour of the MWCNTs/epoxy resin composite strain sensor may also be attributed to the creation of defects, disorders and micro-cracks on the composite^76^.

As listed in Table 6, the 50-BP/PVA composite strain sensor attained the highest sensitivity (2.92 ± 0.05), followed by 65-BP/PVA (2.59 ± 0.05), 80-BP/PVA (1.89 ± 0.05) and 100-BP (0.74 ± 0.05). The smallest gauge factor obtained by 100-BP sample was probably related to the stiffness of MWCNTs buckypaper, which only resulted in a minor change of MWCNTs intrinsic resistance. Regardless, the gauge factor of 100-BP obtained (GF ~ 0.74) was still higher than that of the reported MWCNTs buckypaper, fabricated using vacuum filtration method (GF ~ 0.3482)^77^. The relatively higher gauge factor of BP/PVA composites may be associated with the significant increase in the tunnelling resistance, which is reported to increase exponentially with the inter-particle distance^78^. The increased in tunnelling resistance under mechanical strain may be associated with the increasing of gaps between the MWCNT bundles, which is separated by the electrically insulating PVA matrix. Therefore, the greater the amount of PVA in the composite, the higher the gauge factor of the composite strain sensor obtained. Similarly, according to Hu *et al*.^76^, the electromechanical properties (sensitivity) of the composite were also improved by the increase in tunnelling resistance. In addition, another reason for the higher gauge factor of BP/PVA composites obtained was probably due to the increase in contact resistance due to the decreasing contact areas between adjacent MWCNTs, under stretching. To sum up, the gauge factor of all BP/PVA composites was typically higher than the gauge factor of MWCNTs/polymer composites reported in the literature, which ranged from 0.1 to 1.5^15,45,79–81^.

**Table 6:** Calculated gauge factor of PVA-infiltrated MWCNTs buckypaper samples.

Sample name	Gauge factor calculated
100-BP	0.74 ± 0.05
80-BP/PVA	1.89 ± 0.05
65-BP/PVA	2.59 ± 0.05
50-BP/PVA	2.92 ± 0.05

**Figure 8:**
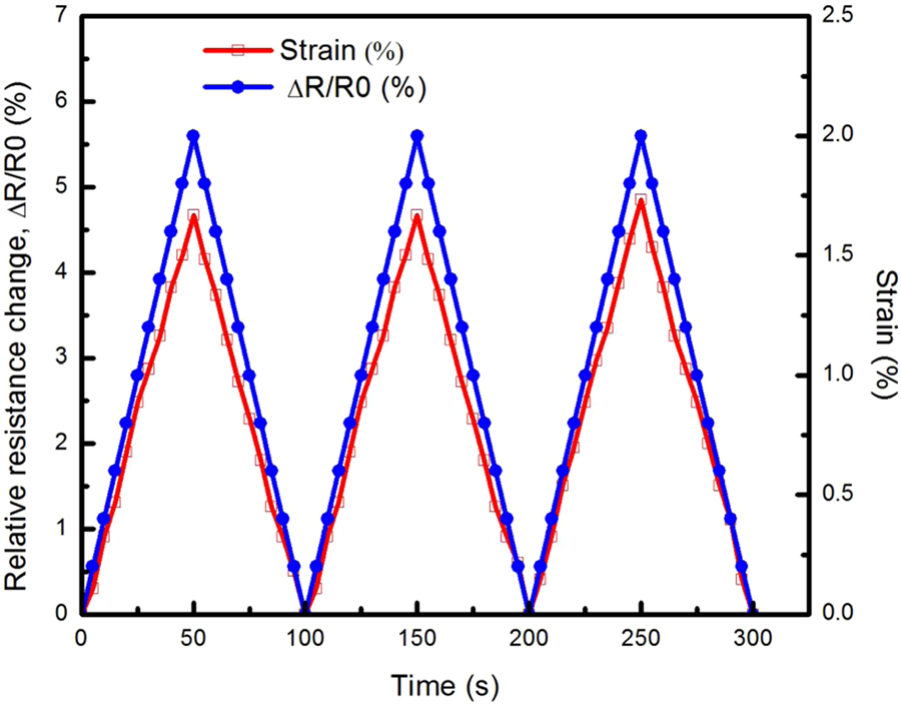
Piezoresistive response of 65-BP/PVA composite under three loading and unloading cycles at 2% strain.

**Figure 9:**
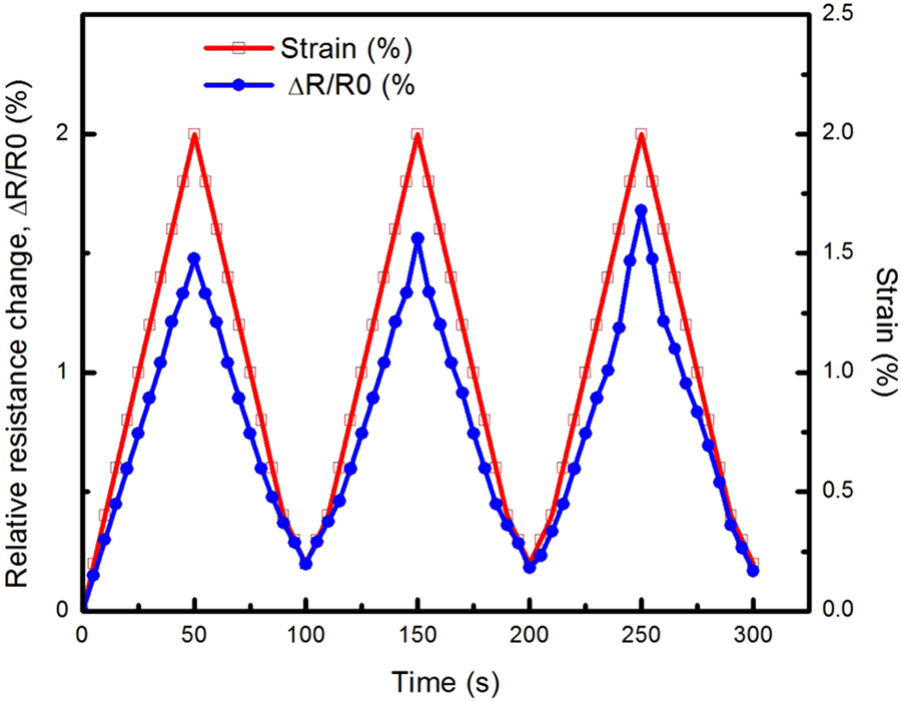
Piezoresistive response of 100-BP sample under three loading and unloading cycles at 2% strain.

To fully unveil the performance of the MWCNTs BP/PVA composites as a strain sensor, a cyclic uniaxial tensile test was conducted to investigate the electromechanical behaviour of the strain sensors during multiple loading-unloading cycles. MWCNTs buckypaper and their composites were tensile-tested under three loading-unloading cycles, within its elastic regime (ε ~ 2%). The relationships of relative resistance change and tensile strain versus time of 65-BP/PVA composite and 100-BP sample are shown in Figs 8 and 9 respectively.

As shown in Fig. 8, the strain was applied by a linear ramp-up to a strain, ε of 2.0%, followed by subsequent unloading to a strain, ε of 0% and then subjected to two repetitions of identical loading-unloading cycles. From the cyclic uniaxial tensile test performed over 300 seconds (Fig. 8), a repeatable nearly-linear response to three loading-unloading cycles was observed, indicating the 65-BP/PVA composite displayed remarkable reproducibility and hysteresis-free operation, in the elastic regime of the strain sensor. This response was maintained for all three cycles without any significant resistance drifting, as no significant phase difference between the piezoresistive response of the composite and the applied load was observed. The hysteresis phenomenon typically occurs in viscoelastic material, which exhibits a time delay in returning to the original shape of the material, due to the energy loss during the loading-unloading cycle. However, in this case, the absence of hysteresis effect in 65-BP/PVA composite was observed in the elastic regime under three consecutive loading-unloading cycle, validating the suitability of BP/PVA composite as a strain sensor. In the elastic regime (ε ~ 2%), the deformation of MWCNTs conductive networks in the composite was assumed to be reversible. After subsequent cyclic tension, the conductive networks stay in the original form and therefore the maximum ΔR/R_0_ can return to their initial value (4.85%). Besides, the strain signal of the 65-BP/PVA composite sensor also returned to zero after completing each consecutive loading-unloading cycle, evidencing the capability of the 65-BP/PVA composite to follow the applied strain under cyclic loading. In a previous study, the relative change in electrical resistance of SWCNTs/PDMS composite showed uniform repeated cyclic trend during the cycling between 2.0 and 5.4% strain^30,82^. Similarly, when subjected to three-cycles of uniaxial tensile loading-unloading, the electrical resistance changes of 65-BP/PVA composite varied in tandem with applied strains. This phenomenon indicated that the 65-BP/PVA composite exhibited linear and reproducible piezoresistive response to the three loading-unloading cycles applied, without destroying the arrangement of MWCNTs networks.

On the other hand, the strain signal of the 100-BP did not revert exactly to zero after each loading-unloading cycle, but remained at a strain of around 0.2%, indicating that the sample was unable to return to its original shape upon unloading. Besides, the maximum ΔR/R_0_ slightly increased after each consecutive loading-unloading cycle (Fig. 9), that may be attributed to the possible hysteresis effect and irreversible destruction of the MWCNTs conductive networks in the buckypaper as a result of permanent deformation. The permanent deformation of the 100-BP sample was caused by an increase in the sample’s original length upon tensile strain unloading. This led to an increase of gap between MWCNTs and a decrease in contact areas between MWCNTs in the network, which resulted in an increase in tunnelling and contact resistances. Therefore, a relatively higher maximum ΔR/R_0_ at both 2^nd^ and 3^rd^ cycle was observed. In summary, the cyclic uniaxial tensile test has validated that the 65-BP/PVA composite displayed a hysteresis-free operation and high reproducibility under 3 loading-unloading cycles, as compared to MWCNTs buckypaper strain sensor (or 100-BP).

In summary, the strain sensing capability (or gauge factor) of BP/polymer nanocomposite was proved to be mainly governed by three major aspects, such as piezoresistivity of strained individual MWCNTs, the contact resistance change due to the increase/decrease of the contact areas between MWCNTs, as well as the tunnelling resistance change between neighbouring MWCNTs due to the change in inter-tube distances^83^. A linear relationship between the relative resistance change and the strain deformation was obtained for all BP/PVA composites, in the elastic range (ε ~ 2.0%). Particularly for 65-BP/PVA composite, a repeatable nearly-linear response to three loading-unloading cycles was observed. This phenomenon indicated that the 65-BP/PVA composite strain sensor demonstrated remarkable reproducibility, in which similar repeated resistance-strain behaviour was observed under cyclic loading-unloading. These behaviours have validated the electromechanical performance of the BP/PVA composites and their suitability for strain sensing application.

## Conclusion

MWCNTs BP/polyvinyl alcohol (PVA) composites were successfully fabricated by a sequence of vacuum filtration and polymer intercalation technique. The PVA infiltration into the porous MWCNTs network enhanced the strain transfer from PVA matrix to MWCNTs in the composite by providing strong interfacial adhesion between the PVA and MWCNTs. The realization of the covalent functionalization of MWCNTs by acid treatment (H_2_SO_4_/HNO_3_) was proved from the FTIR analysis, in which the strongly hydrogen-bonded oxygen-containing functional groups (C=O carbonyl, -OH hydroxyl and -COOH carboxyl moieties) were attached on MWCNTs surface. The optimized conditions for achieving uniform and stable dispersion of MWCNTs suspension was found to be at an ultrasonic amplitude of 54 μm and a sonication time of 40 min, using ethanol as the dispersion medium. The surface modification of MWCNTs buckypaper by infiltrating MWCNTs buckypaper into polyvinyl alcohol (PVA) solution was also successfully achieved. Energy dispersive X-ray (EDX) analysis proved the complete infiltration of PVA solution, as BP/PVA composite exhibited a higher mass fraction of oxygen element (25.95 wt. %) than that of MWCNTs BP (8.72 wt. %). Field emission scanning electron microscopy (FESEM) performed showed that uniformly distributed MWCNTs networks were formed in the composites, forming relatively defect-free composite films.

Finally, the mechanical, electrical and electromechanical properties of the composites with different MWCNTs contents were characterized. MWCNTs BP/PVA composite produced were proved to be more satisfactory for strain sensing application, as compared to MWCNTs buckypaper. Particularly, BP/PVA composite with MWCNTs content of 65 wt.% was found to be the most-suitable composite for strain sensing application. The reasons are the tensile strength, Young’s modulus and elongation-at-break of 65-BP/PVA composite achieved a maximum value of 156.28 MPa, 4.02 GPa and 5.85%, improved by 189%, 443% and 166% respectively, as compared to the MWCNTs BP. 65-BP/PVA composite also showed a significant sensitivity improvement of 250% up to a gauge factor of 2.59. The cyclic uniaxial tensile test proved the high reproducibility and hysteresis-free operation of 65-BP/PVA composite under 3 loading-unloading cycles in the elastic regime (ε ~ 2%). Therefore, 65-BP/PVA composite fabricated was potentially suitable for strain sensing applications, especially in structural health monitoring, wearable technology for human motion detection, as well as can be an alternative choice to the conventional metallic and semiconductor strain sensors.

### Materials

MWCNTs (diameter of 16–23 nm, purity of 98%) were produced from tubular microwave-assisted chemical vapor deposition (TMCVD) method^84^. Both analytical grade concentrated nitric acid, HNO_3_ (70%) and concentrated sulfuric acids, H_2_SO_4_ (98%) were received from Fisher Scientific. EMSURE® ISO standard sodium hydroxide pellets were supplied from Merck Millipore. Poly(vinyl alcohol) PVA powder (99% hydrolyzed) with an average molecular weight of 85,000–124,000 gmol^−1^ was purchased from Sigma Aldrich. Ethanol (99.5%), acetone (99.4%) and methanol (99.8%) were purchased from Systherm Chemicals Malaysia. Hydrophilic polytetrafluoroethylene (PTFE) filter membranes with a pore size of 0.45 µm and a diameter of 47 mm were purchased from Merck Millipore.

## Methods

Fourier-transform infrared (FTIR) analysis was performed using FTIR spectroscopy (PerkinElmer) to identify the functional groups attached to MWCNTs. The IR spectra were collected from 500 to 4000 cm^−1^. The zeta potential of MWCNTs dispersed in different types of solvent were measured using Zetasizer Nano ZS (ZEN3600, Malvern). The microscopic surface features of buckypaper were observed using a scanning electron microscope (FEI Quanta 400 SEM) imaging technique with a magnification of 100,000x at an accelerating voltage of 20 kV. Energy dispersive X-ray (EDX) analysis was performed using the EDX spectroscopy (Oxford-Instruments INCA 400 with X-Max Detector). For mechanical characterization, the tensile performance of rectangular-shaped MWCNTs buckypaper samples (20 mm × 5 mm) was evaluated using a computerized tensile test machine (Lloyd LR10K Plus). The specimen geometry and dimensions were selected by rescaling the specimen type 2 recommended by the ISO 527-3 standard, similar to ASTM D-882 standard. A constant extension rate of 0.1 mm/min was applied until the fracture of films occurred. For the electrical characterization, two copper conductive strips were attached at the opposite ends of buckypaper samples using silver adhesive to minimize contact resistance, with a gauge length of 15 mm. DC electric resistance measurement was carried out with a digital multimeter (Sanwa Japan CD800a) using two-point probe method, in accordance to ASTM D4496-13 standard. The surface resistivity (ρ_s_) and bulk resistivity (ρ_b_) were calculated using the following Eqs 2 and 3.

2 $$\rho s=\frac{R\times W}{L}$$

3 $${\rho }_{{\rm{b}}}=\frac{R\times W\times t}{L}$$

The thickness of MWCNTs based films was measured using a digital display micrometer (Mitutoyo Japan). Finally, the corresponding electrical conductivity, σ was calculated using Eq. 4.

4 $$\sigma =\frac{1}{{\rho }_{{\rm{b}}}}$$

The electrical properties (electron density, electron mobility, resistivity and conductivity) of the MWCNTs BP samples were further investigated by a four-point probe technique using a van der Pauw Hall measurement system (Ecopia HMS 3000, Korea), applied under magnetic field at a temperature of 300 K. By sourcing a known direct current, I of 15 mA through the outer probes and measuring the resulting voltage drop ∆V, the resistance of the samples can also be determined by applying Ohm’s Law (R = ∆V/I). For electromechanical characterization, the sensitivity of the strain sensor is quantified as a gauge factor (GF), expressed as the ratio of relative change in electrical resistance to applied strain, as shown in Eq. 5.

5 $${\rm{GF}}=\frac{{\rm{\Delta }}{\rm{R}}/\mathrm{Ro}}{{\rm{\varepsilon }}}$$

The change in resistance of buckypaper samples was constantly measured using a digital multimeter (Agilent 34401 A) as a function of strain applied. The gauge factor was measured in the linear elastic deformation zone (ε ~ 2%). The cyclic uniaxial tensile test was performed by employing three loading-unloading cycles in the linear elastic region (ε ~ 2%) and the relative resistance change as a function of mechanical strain was measured over a period of 300 s. The characterizations were repeated three times for each type of strain sensor to ensure the consistency and reproducibility of data.

### Preparation of functionalized MWCNTs

MWCNTs were immersed in a mixture of concentrated H_2_SO_4_ (98%)/HNO_3_ (70%) (3:1 v/v) to introduce oxygenated groups on the surface of the MWCNTs through covalent functionalization. The mixture was bath-sonicated under the mild acidic condition for 3 hr at room temperature. Bath sonication in combination with acid treatment was performed to create defects in the carbon lattice so that the carboxyl groups can attach to the surface of MWCNTs. Simultaneously, the treatment also purifies the MWCNTs by removing any residual metallic catalyst. The acidic mixture was poured into a large beaker containing distilled water. Sodium hydroxide was added to neutralize the strong acidic conditions of the mixture. The mixture was left overnight for the settling of MWCNTs under gravity. The supernatant was removed and the remaining mixture was filtered using a PTFE filter membrane. Oxidized MWCNTs attached on the surface of PTFE filter membrane were rinsed repeatedly with deionized water until a neutral solution with pH of around 7 is obtained. Oxidized MWCNTs were dried overnight in a vacuum oven (Tech-Lab Scientific, TVAC-92) at 60 °C to remove all water content.

### Preparation of oxidized MWCNTs buckypaper

400 mg oxidized MWCNTs were initially mixed in 50 ml ethanol. The suspension was dispersed using low power ultrasonic bath cleaner for 1 hour, followed by high power (500 W, 20 kHz) probe-ultrasonication for 40 min at 60% ultrasonic amplitude (54 μm) using a probe sonicator (SonoMechanics, LSP-500). Pulse mode of probe-sonicator was selected, in which sonication energy was applied to the MWCNTs suspension at 15 sec intervals to prevent overheating. Few drops of methanol are added to further promote the hydrophilicity of the PTFE filter membrane, by opening up the pore of the membrane. The uniformly dispersed mixture was then filtrated through the hydrophilic PTFE filter membrane using a diaphragm type vacuum pump (ULVAC DTC-41). After the filtration process, MWCNTs buckypaper was repeatedly washed with deionized water to remove any adsorbed methanol on the buckypaper surface. Finally, MWCNTs buckypaper samples were carefully peeled off from the PTFE filter membrane. Free-standing MWCNTs buckypaper samples were dried in a vacuum oven for 24 h at 60 °C and were weighed to determine the corresponding mass of buckypaper.

### Preparation of PVA-infiltrated MWCNT buckypaper

The choice of a polymer containing suitable functional groups is vital to develop strong interfacial interaction between the polymer matrix and MWCNTs filler. Among different kinds of polymers, water-soluble synthetic polymer, poly(vinyl alcohol) (PVA) with a chemical formula of (C_2_H_4_O)_n_ is an ideal candidate to be used as the composite matrix, due to the biodegradable and low-cost advantage. Pre-experimental testing was conducted to ensure that the viscosity of the PVA solution prepared was not too high and no residues were formed. A clear 10 wt. % liquid PVA solution is prepared by dissolving PVA powder in deionized water under bath sonication (25 °C) for 30 min and mechanical stirring (600 rpm, 120 °C) for 2 hr, in an alternating sequence.

As-prepared MWCNTs buckypaper was temporarily soaked in pure acetone to render the MWCNTs buckypaper hydrophilic and to facilitate the formation of hydrogen bonds with the PVA solution. To prepare MWCNTs BP/PVA composites with different MWCNTs loadings, MWCNTs buckypaper fabricated were impregnated with different amounts of 10 wt. % PVA solution for a period of 24 h to ensure that the buckypaper was completely infiltrated with PVA solution. Subsequently, PVA-infiltrated MWCNTs buckypaper with different weight loadings were dried overnight in a vacuum oven at 60 °C. The weight fraction of MWCNTs in the BP/PVA composite samples was calculated by dividing the mass of the dried MWCNTs buckypaper with the mass of the BP/PVA composite samples. The final samples were designated as *x-BP* (without polymer infiltration) and *x-BP/PVA* (with polymer infiltration), whereby *x* is the weight percentage of MWCNTs in the composite.

## References

[1] Iijima S, Ichihashi T (1993). Single-shell carbon nanotubes of 1-nm diameter. nature.

[2] Xie X-L, Mai Y-W, Zhou X-P (2005). Dispersion and alignment of carbon nanotubes in polymer matrix: A review. Materials Science and Engineering: R: Reports.

[3] Peigney A, Laurent C, Flahaut E, Bacsa RR, Rousset A (2001). Specific surface area of carbon nanotubes and bundles of carbon nanotubes. Carbon.

[4] Bandaru PR (2007). Electrical properties and applications of carbon nanotube structures. Journal of nanoscience and nanotechnology.

[5] Aqel A, El-Nour KMA, Ammar RA, Al-Warthan A (2012). Carbon nanotubes, science and technology part (I) structure, synthesis and characterisation. Arabian Journal of Chemistry.

[6] Wang X, Sparkman J, Gou J (2017). Strain sensing of printed carbon nanotube sensors on polyurethane substrate with spray deposition modeling. Composites Communications.

[7] Suzuki K (2016). Rapid-Response, Widely Stretchable Sensor of Aligned MWCNT/Elastomer Composites for Human Motion Detection. ACS Sensors.

[8] Torres, R. *et al*. *Strain Sensor Based on MWCNT-natural Rubber Composite for Wearable Electronics*. (2016).

[9] Khan Faiza, Kausar Ayesha, Siddiq Muhammad (2015). Polyvinylchloride intercalated poly(ethylene glycol)-modified-multi-walled carbon nanotube buckypaper composites via resin-infiltration technique. Journal of Plastic Film & Sheeting.

[10] M. F. Lima, A., Castro, V., Borges, R. & Silva, G. *Electrical Conductivity and Thermal Properties of Functionalized Carbon Nanotubes/Polyurethane Composites*. Vol. 22 (2011).

[11] Frizzell, C. J. *et al*. *Reinforcement of macroscopic carbon nanotube structures by polymer intercalation: The role of polymer molecular weight and chain conformation*. Vol. 72 (2005).

[12] Sijimol MR (2015). Review on Fate, Toxicity, and Remediation of Perchlorate. Environmental Forensics.

[13] Pham GT, Park Y-B, Liang Z, Zhang C, Wang B (2008). Processing and modeling of conductive thermoplastic/carbon nanotube films for strain sensing. Composites Part B: Engineering.

[14] Oliva-Avilés AI, Avilés F, Sosa V (2011). Electrical and piezoresistive properties of multi-walled carbon nanotube/polymer composite films aligned by an electric field. Carbon.

[15] Bautista-Quijano JR, Avilés F, Aguilar JO, Tapia A (2010). Strain sensing capabilities of a piezoresistive MWCNT-polysulfone film. Sensors and Actuators A: Physical.

[16] Hu N (2013). Ultrasensitive strain sensors made from metal-coated carbon nanofiller/epoxy composites. Carbon.

[17] Bouhamed A, Müller C, Choura S, Kanoun O (2017). Processing and characterization of MWCNTs/epoxy nanocomposites thin films for strain sensing applications. Sensors and Actuators A: Physical.

[18] Njuguna MK, Yan C, Hu N, Bell JM, Yarlagadda PKDV (2012). Sandwiched carbon nanotube film as strain sensor. Composites Part B: Engineering.

[19] Wu TM, Chen EC, Lin YW, Chiang MF, Chang GY (2008). Preparation and characterization of melt‐processed polycarbonate/multiwalled carbon nanotube composites. Polymer Engineering & Science.

[20] Fan Q (2012). The use of a carbon nanotube layer on a polyurethane multifilament substrate for monitoring strains as large as 400%. Carbon.

[21] Miculescu M, Thakur VK, Miculescu F, Voicu SI (2016). Graphene-based polymer nanocomposite membranes: a review. Polymers for Advanced Technologies.

[22] Garlof S (2016). Electro-mechanical piezoresistive properties of three dimensionally interconnected carbon aerogel (Aerographite)-epoxy composites. Composites Science and Technology.

[23] Li J, Yue W, Qin W, Wang C (2017). Approach to controllable tribological properties of sintered polycrystalline diamond compact through annealing treatment. Carbon.

[24] Atif R, Inam F (2016). Reasons and remedies for the agglomeration of multilayered graphene and carbon nanotubes in polymers. Beilstein journal of nanotechnology.

[25] Madhumitha G, Fowsiya J, Mohana Roopan S, Thakur VK (2018). Recent advances in starch–clay nanocomposites. International Journal of Polymer Analysis and Characterization.

[26] Dai H, Thostenson ET, Schumacher T (2015). Processing and characterization of a novel distributed strain sensor using carbon nanotube-based nonwoven composites. Sensors.

[27] Makireddi S, Shivprasad S, Kosuri G, Varghese FV, Balasubramaniam K (2015). Electro-elastic and piezoresistive behavior of flexible MWCNT/PMMA nanocomposite films prepared by solvent casting method for structural health monitoring applications. Composites Science and Technology.

[28] Ramalingame R (2016). MWCNT-PDMS Nanocomposite Based Flexible Multifunctional Sensor for Health Monitoring. Procedia Engineering.

[29] Garg P (2011). Effect of dispersion conditions on the mechanical properties of multi-walled carbon nanotubes based epoxy resin composites. Journal of Polymer Research.

[30] Yamada Takeo, Hayamizu Yuhei, Yamamoto Yuki, Yomogida Yoshiki, Izadi-Najafabadi Ali, Futaba Don N., Hata Kenji (2011). A stretchable carbon nanotube strain sensor for human-motion detection. Nature Nanotechnology.

[31] Zhang Z, Wei H, Liu Y, Leng J (2015). Self-sensing properties of smart composite based on embedded buckypaper layer. Structural Health Monitoring.

[32] Shindo Y, Kuronuma Y, Takeda T, Narita F, Fu S-Y (2012). Electrical resistance change and crack behavior in carbon nanotube/polymer composites under tensile loading. Composites Part B: Engineering.

[33] Inam F, Bhat BR, Vo T, Daoush WM (2014). Structural health monitoring capabilities in ceramic–carbon nanocomposites. Ceramics International.

[34] Luo, W., Liu, Y. & Saha, M. CNT Bucky Paper Enhanced Sandwich Composites for *In-Situ* Load Sensing. V014T011A044, (2017).

[35] Muhulet, A. *et al*. Fundamentals and scopes of doped carbon nanotubes towards energy and biosensing applications. **9**, 154–186, (2018).

[36] Oh H-J, Omori H, Sadakata M, Tsubokura I, Isono Y (2014). Characterization of Interlayer Sliding Deformation for Individual Multiwalled Carbon Nanotubes Using Electrostatically Actuated Nanotensile Testing Device. Journal of Microelectromechanical Systems.

[37] Kim H-I (2017). Tensile properties of millimeter-long multi-walled carbon nanotubes. Scientific reports.

[38] Nakano T, Okamoto Y (2001). Synthetic helical polymers: conformation and function. Chemical Reviews.

[39] Begum A, Tripathi KM, Sarkar S (2014). Water-Induced Formation, Characterization, and Photoluminescence of Carbon Nanotube-Based Composites of Gadolinium(III) and Platinum(II) Dithiolenes. Chemistry – A European Journal.

[40] Li Z, Liang Z (2017). Optimization of Buckypaper-enhanced Multifunctional Thermoplastic Composites. Scientific Reports.

[41] Han J-H, Zhang H, Chu P-F, Imani A, Zhang Z (2015). Friction and wear of high electrical conductive carbon nanotube buckypaper/epoxy composites. Composites Science and Technology.

[42] Han J-H, Zhang H, Chen M-J, Wang G-R, Zhang Z (2014). CNT buckypaper/thermoplastic polyurethane composites with enhanced stiffness, strength and toughness. Composites Science and Technology.

[43] Pham Giang T, Park Young-Bin, Wang Shiren, Liang Zhiyong, Wang Ben, Zhang Chuck, Funchess Percy, Kramer Leslie (2008). Mechanical and electrical properties of polycarbonate nanotube buckypaper composite sheets. Nanotechnology.

[44] Tripathi KM, Begum A, Sonkar SK, Sarkar S (2013). Nanospheres of copper(iii) 1,2-dicarbomethoxy-1,2-dithiolate and its composite with water soluble carbon nanotubes. New Journal of Chemistry.

[45] Wichmann MHG, Buschhorn ST, Gehrmann J, Schulte K (2009). Piezoresistive response of epoxy composites with carbon nanoparticles under tensile load. Physical Review B.

[46] Leung, M. A. A. S. N. In *SPE ANTEC* (Indianapolis, 2016).

[47] Stobinski L (2010). Multiwall carbon nanotubes purification and oxidation by nitric acid studied by the FTIR and electron spectroscopy methods. Journal of Alloys and Compounds.

[48] Morsy M, Helal M, El-Okr M, Ibrahim M (2014). Preparation, purification and characterization of high purity multi-wall carbon nanotube. Spectrochim Acta A Mol Biomol Spectrosc.

[49] Mallakpour S, Abdolmaleki A, Borandeh S (2014). l-Phenylalanine amino acid functionalized multi walled carbon nanotube (MWCNT) as a reinforced filler for improving mechanical and morphological properties of poly (vinyl alcohol)/MWCNT composite. Progress in Organic Coatings.

[50] Alghunaim NS (2016). Optimization and spectroscopic studies on carbon nanotubes/PVA nanocomposites. Results in Physics.

[51] Salam MA, Burk R (2017). Synthesis and characterization of multi-walled carbon nanotubes modified with octadecylamine and polyethylene glycol. Arabian Journal of Chemistry.

[52] Gonçalves B (2017). Development of water-based printable piezoresistive sensors for large strain applications. Composites Part B: Engineering.

[53] Tao, M. *et al*. In *MATEC Web of Conferences*. 06072 (EDP Sciences).

[54] Lee J, Kim M, Hong CK, Shim SE (2007). Measurement of the dispersion stability of pristine and surface-modified multiwalled carbon nanotubes in various nonpolar and polar solvents. Measurement Science and Technology.

[55] Parveen S, Rana S, Fangueiro R, Paiva MC (2017). Characterizing dispersion and long term stability of concentrated carbon nanotube aqueous suspensions for fabricating ductile cementitious composites. Powder Technology.

[56] Kharisov, B. *Dispersion of carbon nanotubes in water and non-aqueous solvent*s. (2013).

[57] Xu G, Zhang Q, Zhou W, Huang J, Wei F (2008). The feasibility of producing MWCNT paper and strong MWCNT film from VACNT array. Applied Physics A: Materials Science & Processing.

[58] Motta M. S., Moisala A., Kinloch I. A., Windle A. H. (2008). The Role of Sulphur in the Synthesis of Carbon Nanotubes by Chemical Vapour Deposition at High Temperatures. Journal of Nanoscience and Nanotechnology.

[59] Cha JE, Kim SY, Lee SH (2016). Effect of Continuous Multi-Walled Carbon Nanotubes on Thermal and Mechanical Properties of Flexible Composite Film. Nanomaterials.

[60] Nezhad HY, Thakur VK (2018). Effect of Morphological Changes due to Increasing Carbon Nanoparticles Content on the Quasi-Static Mechanical Response of Epoxy Resin. Polymers.

[61] Malik S (2004). Failure mechanism of free standing single-walled carbon nanotube thin films under tensile load. Physical Chemistry Chemical Physics.

[62] Mallakpour S, Dinari M (2013). The synergetic effect of chiral organoclay and surface modified-Al2O3 nanoparticles on thermal and physical properties of poly(vinyl alcohol) based nanocomposite films. Progress in Organic Coatings.

[63] Ni W, Wang B, Wang H, Zhang Y (2006). Fabrication and properties of carbon nanotube and poly (vinyl alcohol) composites. Journal of Macromolecular Science, Part B.

[64] Lin Jia-Horng, Lin Zheng-Ian, Pan Yi-Jun, Hsieh Chien-Teng, Huang Chien-Lin, Lou Ching-Wen (2016). Thermoplastic polyvinyl alcohol/multiwalled carbon nanotube composites: Preparation, mechanical properties, thermal properties, and electromagnetic shielding effectiveness. Journal of Applied Polymer Science.

[65] Yee KF, Ong YT, Mohamed AR, Tan SH (2014). Novel MWCNT-buckypaper/polyvinyl alcohol asymmetric membrane for dehydration of etherification reaction mixture: Fabrication, characterisation and application. Journal of Membrane Science.

[66] Dominiczak M (2011). Evaluation of the nanotube intrinsic resistance across the tip-carbon nanotube-metal substrate junction by Atomic Force Microscopy. Nanoscale research letters.

[67] Xu H, Chen L, Hu L, Zhitenev N (2010). Contact resistance of flexible, transparent carbon nanotube films with metals. Applied Physics Letters.

[68] Kumar V, Haspel H, Nagy K, Rawal A, Kukovecz A (2016). Leveraging compressive stresses to attenuate the electrical resistivity of buckypaper. Carbon.

[69] Wang D, Song P, Liu C, Wu W, Fan S (2008). Highly oriented carbon nanotube papers made of aligned carbon nanotubes. Nanotechnology.

[70] DeGraff J (2017). Printable low-cost and flexible carbon nanotube buckypaper motion sensors. Materials & Design.

[71] Moreno Marcelino JE, Vigueras Santiago E, Lopez-Tellez G, Hernández López S (2014). Chemical Functionalization of Carbon Nanotubes and its Effects on Electrical Conductivity. Journal of Nano Research.

[72] Thakur, S. & Hu, J. In *Aspects of Polyurethanes* (InTech, 2017).

[73] Baumgartner G (1997). Hall effect and magnetoresistance of carbon nanotube films. Physical Review B.

[74] Liu L (2000). Controllable Reversibility of an sp 2 to sp 3 Transition of a Single Wall Nanotube under the Manipulation of an AFM Tip: A Nanoscale Electromechanical Switch?. Physical Review Letters.

[75] Tadakaluru S, Thongsuwan W, Singjai P (2014). Stretchable and Flexible High-Strain Sensors Made Using Carbon Nanotubes and Graphite Films on Natural Rubber. Sensors (Basel, Switzerland).

[76] Hu N (2010). Investigation on sensitivity of a polymer/carbon nanotube composite strain sensor. Carbon.

[77] Vemuru S, Wahi R, Nagarajaiah S, Ajayan P (2009). Strain sensing using a multiwalled carbon nanotube film. The Journal of Strain Analysis for Engineering Design.

[78] Rein MD, Breuer O, Wagner HD (2011). Sensors and sensitivity: Carbon nanotube buckypaper films as strain sensing devices. Composites Science and Technology.

[79] Loh KJ, Lynch JP, Shim B, Kotov N (2008). Tailoring piezoresistive sensitivity of multilayer carbon nanotube composite strain sensors. Journal of Intelligent Material Systems and Structures.

[80] Amjadi M, Yoon YJ, Park I (2015). Ultra-stretchable and skin-mountable strain sensors using carbon nanotubes–Ecoflex nanocomposites. Nanotechnology.

[81] Giffney T, Bejanin E, Kurian AS, Travas-Sejdic J, Aw K (2017). Highly stretchable printed strain sensors using multi-walled carbon nanotube/silicone rubber composites. Sensors and Actuators A: Physical.

[82] Tripathi KM, Vincent F, Castro M, Feller JF (2016). Flax fibers – epoxy with embedded nanocomposite sensors to design lightweight smart bio-composites. Nanocomposites.

[83] Bao W, Meguid S, Zhu Z, Weng G (2012). Tunneling resistance and its effect on the electrical conductivity of carbon nanotube nanocomposites. Journal of Applied Physics.

[84] Mubarak NM, Sahu JN, Abdullah EC, Jayakumar NS, Ganesan P (2014). Single stage production of carbon nanotubes using microwave technology. Diamond and Related Materials.

